# Muscle activation patterns during backward walking in people with chronic ankle instability

**DOI:** 10.1186/s12891-020-03512-x

**Published:** 2020-07-25

**Authors:** Tharani Balasukumaran, Uri Gottlieb, Shmuel Springer

**Affiliations:** Faculty of Health Sciences, Department of Physical Therapy, Ariel University, Ariel, Israel

**Keywords:** Chronic ankle instability, Walking, Electromyography

## Abstract

**Background:**

Altered walking patterns are often described in individuals with chronic ankle instability (CAI). Contemporary treatment paradigms recommend backward walking (BW) to improve locomotion in people with musculoskeletal disorders. The purpose of this study was to determine whether muscle activity and activation variability during BW differs between subjects with and without CAI.

**Methods:**

Sixteen participants with CAI and 16 healthy controls walked on a treadmill at their self-selected speed under BW and forward walking (FW) conditions. Surface electromyography (EMG) data for the peroneus longus, tibialis anterior, medial gastrocnemius and gluteus medius muscles were collected. EMG amplitude normalized to maximum voluntary isometric contraction (%MVIC) and the standard deviation (SD) of the %MVIC EMG amplitude was calculated throughout the gait cycle. In addition, the area under the curve (AUC) of the %MVIC EMG amplitude was calculated before and after initial contact (pre-IC: 90–100% of stride; post-IC: 0–10% of stride).

**Results:**

No differences between groups were noted in the %MVIC amplitude or activation variability (SD of %MVIC EMG) under BW or FW. In both groups, decreased tibialis anterior (*p* < 0.001) and gluteus medius (*p* = 0.01), and increased medial gastrocnemius (*p* < 0.001) activation were observed during pre- and post-IC under BW condition.

**Conclusion:**

Participants with CAI and healthy controls have similar muscle activity patterns during BW. Yet, the results should be interpreted with caution due to the heterogeneity of the CAI population.

## Background

Ankle sprains are among the most common musculoskeletal injuries [1, 2]. Up to 59% of individuals with previous history of an ankle sprain develop chronic ankle instability (CAI) [3]. CAI is a function diminishing condition characterized by repetitive episodes or perception of the ankle giving way. It may be accompanied by ongoing symptoms such as pain, weakness, and reduced ankle range of motion [4, 5]. When compared with healthy participants, individuals with CAI display deficits in sensorimotor control, such as impaired sense of joint position [6], muscle weakness due to arthrogenic muscles inhibition [7] and decreased postural control [8].

The precise analysis of electromyography (EMG) muscle activity during movement is an essential step in understanding sensorimotor control. Studies that assessed amplitude of muscle activity during walking reported differences between individuals with and without CAI. While walking, participants with CAI were reported to have increased peroneus longus activity before and after initial contact (IC) [9–11], lower tibialis anterior and greater medial gastrocnemius muscle activation before IC [11], and increased gluteus medius muscle activation amplitude during late stance through early swing phase [11]. However, other studies did not find differences in the amplitude of muscle activation between the CAI and control groups [12, 13].

Another factor that seems to be affected by CAI is the variability of muscle function during walking. Up to date, two main techniques were used for assessing variability of muscle function during walking in subjects with CAI. Kautzky et al. [14] utilize traditional linear variability measures such as, the standard deviation (SD) of timing of muscle activation relative to IC (quantified in milliseconds), the SD of percent of muscle activation time across the stride cycle, and the coefficient of variation (COV) of activation amplitude before and after IC. Individuals with CAI had more variable time of activation of the biceps femoris relative to IC, as well as, increased variability of gluteus medius EMG maximal amplitude prior to IC compared to a healthy group. Koldenhoven and colleagues [15] provided more informative and dynamic presentation of muscle variability by plotting the SD of the EMG amplitude across the entire gait cycle and used it as the primary outcome to present variability. Compared to healthy controls, CAI group had decreased variability in muscle activation amplitude of the peroneus longus from 1 to 10%, 32–38% and 56–100% of the gait cycle, as well as reduced tibialis anterior variability from 33 to 42%, 57–69%, and 95–99% of the gait cycle. Altered EMG amplitude variability may indicate a constraint in the adaptability of the sensorimotor system. Furthermore, it has been shown that challenging walking conditions, such as walking at fast speeds, emphasize differences in muscle activity patterns between healthy individuals and those with CAI [13].

Contemporary treatment paradigms for altered gait in people with CAI recommend gait training [16]. Backward walking (BW) is a gait activity with additional complexity compared to regular forward walking (FW), that may be novel and challenging task even for healthy individuals. Evidence suggests that BW utilizes additional elements, presumably supraspinal, in addition to a common spinal drive of gait [17, 18]. Kurz et al. [19] reported increase in sensorimotor cortical activation measured by functional near infrared spectroscopy in healthy adults during BW. It has also been reported that exercise of BW in untrained healthy adults caused neural adaptations [19]. As BW requires more central nervous system resources than does FW, it may serve as a training method to enhance sensorimotor control of movement among individuals with CAI.

BW has indeed been shown to improve control of movement in patients with neurological lesions and with musculoskeletal disorders. A recent study reported the effectiveness of BW as a rehabilitation technique for improving knee proprioception in patients after anterior cruciate ligament reconstruction [20]. The beneficial effects of BW have also been demonstrated in people with patellofemoral pain syndrome [21], and in with low back pain [22].

Studies that assessed muscle firing patterns in BW compared to FW indicated that it is not a simple reversal of the EMG pattern of FW [23, 24]. The EMG activity of the rectus femoris and vastus medialis were considerably higher when walking backward rather than forward [25, 26]. Significant differences were also found in the activation of the ankle muscles. Both ankle flexors and extensors are co-activated at early stance during FW, compared to activation of the ankle flexors only during BW [27]. Moreover, it was found that the mean EMG activity of the gluteus medius and tibialis anterior throughout the gait cycle is higher in BW than in FW [27].

The current study investigated the effects of forward and backward walking conditions on EMG amplitude and variability of the peroneus longus, tibialis anterior, medial gastrocnemius and gluteus medius muscles among subjects with and without CAI. We hypothesized that during BW, compared to healthy controls, individuals with CAI would have increased amplitude and activation variability of peroneus longus and tibialis anterior muscles, as measured by EMG.

## Methods

The study was approved by the University Institutional Review Board approved the study, approval number: AU-HEA-20190213. All participants provided written informed consent.

### Participants

The study sample included 16 participants with CAI and 16 aged-matched healthy controls. The inclusion criteria for the CAI group were in accordance with The International Ankle Consortium recommendations [4, 28]: (i) history of at least one significant ankle sprain that occurred at least 12 months prior to the study, characterized by inflammatory symptoms and caused at least 1 day of decreased physical activity, (ii) history of at least two episodes of ‘giving way’ and feelings of ankle joint instability during the last 6 months, (iii) the most recent acute ankle sprain occurred more than 3 months prior to study enrollment, (iv) a positive response to at least five yes/no questions (question 1, plus four others) of the Ankle Instability Instrument [5, 28].

The control group included healthy participants with no history of ankle sprain. Exclusion criteria from both groups were history of ankle fracture, other pathological conditions or surgical procedures in the lower extremity within 1 year of study participation or had vestibular or neurological disorders.

### Procedure

The study was conducted during one visit at the Neuromuscular and Human Performance Laboratory, at XXX University. EMG activity of the peroneus longus, tibialis anterior, medial gastrocnemius and gluteus medius muscles was evaluated under both FW and BW walking conditions, while participants walked barefoot on a treadmill. Participants were instructed to walk at a comfortable, self-selected pace. Before data collection, they were provided with an opportunity to habituate to forward and backward walking on the treadmill. At this time, walking speed was adjusted according to the participant’s comfort. This self-selected, comfortable speed during forward and backward walking was used during the gait assessments. EMG data were collected using a wireless EMG system (Delsys Trigno, Delsys Inc., Boston, MA) at 2000 Hz. Before data collection, participants’ skin was shaved and cleaned with isopropyl alcohol to minimize impedance. Electrode placement was performed according to Surface Electromyography for the Non-Invasive Assessment of Muscles (SENIAM) guidelines [29]. Lightweight (< 15 g) wireless rectangular sensors (37x26x15mm) with parallel-bar silver contact electrodes were placed over the muscle belly of the recorded muscle using a double-sided adhesive interface (Delsys Inc., Boston, MA). The Delsys EMG senor’s range is 11 mV, its resolution is 168 nV/bit, and the overall channel noise is < 0.75uV. Before walking, EMG activity during maximal voluntary isometric contraction (MVIC) was measured against manual resistance for all recorded muscles. Visual inspection was made prior to data collection to minimize crosstalk. It should be also noted that Delsys sensors are designed with an interface and inter-electrode distance to offer the optimal crosstalk suppression while maintaining the EMG signal amplitude. Each MVIC was performed three times for 5 s, with 60 s rest between contractions.

The raw EMG signal was filtered through band pass filter between 50 and 500 Hz with the use of a fourth-order Butterworth filter, and processed with EMG works software (version 4.1.1, Delsys, Boston, MA). MVIC EMG signals were subjected to a root mean square (RMS) algorithm to convert all amplitude measures to positive values. Gait events were identified using inertial measurement sensors that were placed near the participant’s heel during FW and near the second metatarsal head during BW. Gait cycles were isolated by identifying consecutive heel or toes strikes. The vertical accelerometer signal from the sensor placed near the heel (for FW) or second metatarsal head (for BW) was used to identify initial contact. A foot contact was characterized as a rapid change in vertical acceleration signal followed by a rather lengthy steady-state period [30]. Subjects walked barefoot on a motorized treadmill (VO2 Challenger, Taiwan). The condition order was fixed, with FW being recorded first, following by BW walking. Fifteen consecutive strides from the beginning of each walking condition were analyzed.

The EMG amplitudes during the gait cycle were transformed to 100 data points for each of the 15 consecutive strides, and their output was displayed as a percentage of the MVIC EMG value (%MVIC), which can be used to establish a common ground when comparing data between participants. In addition, to assess stride-to-stride variability of muscle activity, the standard deviation (SD) of the EMG amplitude (as %MVIC) was calculated for each participant at each of the 100 data points. The %MVIC served as the dependent variable for comparing EMG amplitude across the entire gait cycle between groups, whereas the SD of %MVIC served as the dependent variables for comparing activation variability across the entire gait cycle between groups. Finally, the area under the curve (AUC) of the muscles’ activation (%MVIC) was calculated before and after IC (pre-IC; post-IC) during both walking conditions. Pre-IC was defined as 90–100% of the gait cycle, and post-IC was defined as 0–10% of the gait cycle.

These periods during the gait cycle have previously been demonstrated to be relevant to the study of gait dynamics in people with CAI [14, 15]. The AUC during Pre-IC and Post-IC served as the dependent variable for comparing the adaptation of each group from FW to BW.

### Statistical analysis

Descriptive statistics included mean (M) and standard deviations (SD). Simple chi-square and independent t-tests were used to compare baseline characteristics between the CAI and control groups.

For between-groups analysis, under each walking condition, group mean muscle amplitude activity (%MVIC) and EMG variability (presented as SD) with their respective 95% confidence interval (CI) were plotted for each muscle throughout the gait cycle [31, 32]. A significant, meaningful difference was defined in case a non-overlapping CI was found for consecutive 3% of the stride [15, 33]. According to this method, post-hoc analysis was conducted only when significant between-groups differences in muscle mean amplitude activation or SD was found.

To compare the adaptation of each group to BW, a linear mixed model with group and condition as fixed effects and participants as random effect was conducted to examine the effect of group and condition on the AUC during Pre -and Post-IC of each muscle separately. In addition, EMG amplitude difference between FW and BW was calculated (FW – BW) for each muscle and plotted with the respective 95% CI. As previously described, non-overlapping CIs for more than 3% of the gait cycle was considered as significant difference in adaptation. A significance level of < 0.05 was set. Analysis was conducted using IBM SPSS Statistics for Windows, Version 24.0 (IBM Corp, Armonk, NY).

## Results

### Participant characteristics

Participant characteristics are presented in Table 1. There were no differences in baseline characteristics (age, height, weight and sex) between groups.

**Table 1:** Participant characteristics

Parameter	CAI	Control	*p*-value
Age (years)	25.44 (2.39)	25.56 (3.44)	0.57
Height (m)	1.71 (0.11)	1.72 (0.10)	0.82
Weight (kg)	71.69 (13.82)	68.36 (12.44)	0.40
Sex (F/M)	8F/8M	9F/7M	0.72
Forward walking speed (m/sec)	1.05 (0.16)	1.12 (0.10)	0.14
Backward walking speed (m/sec)	0.62 (0.14)	0.63 (0.15)	0.65
Ankle with recurrent sprains (RT/LT)	14/2	–	–
Time since last sprain (weeks)	20.5 (18.18)	–	–
Ankle Instability Instrument score	6 (1.15)	–	–

*CAI* Chronic ankle instability, *RT* Right, *LT* Left

### EMG amplitude activity across the cycle

**Fig. 1:**
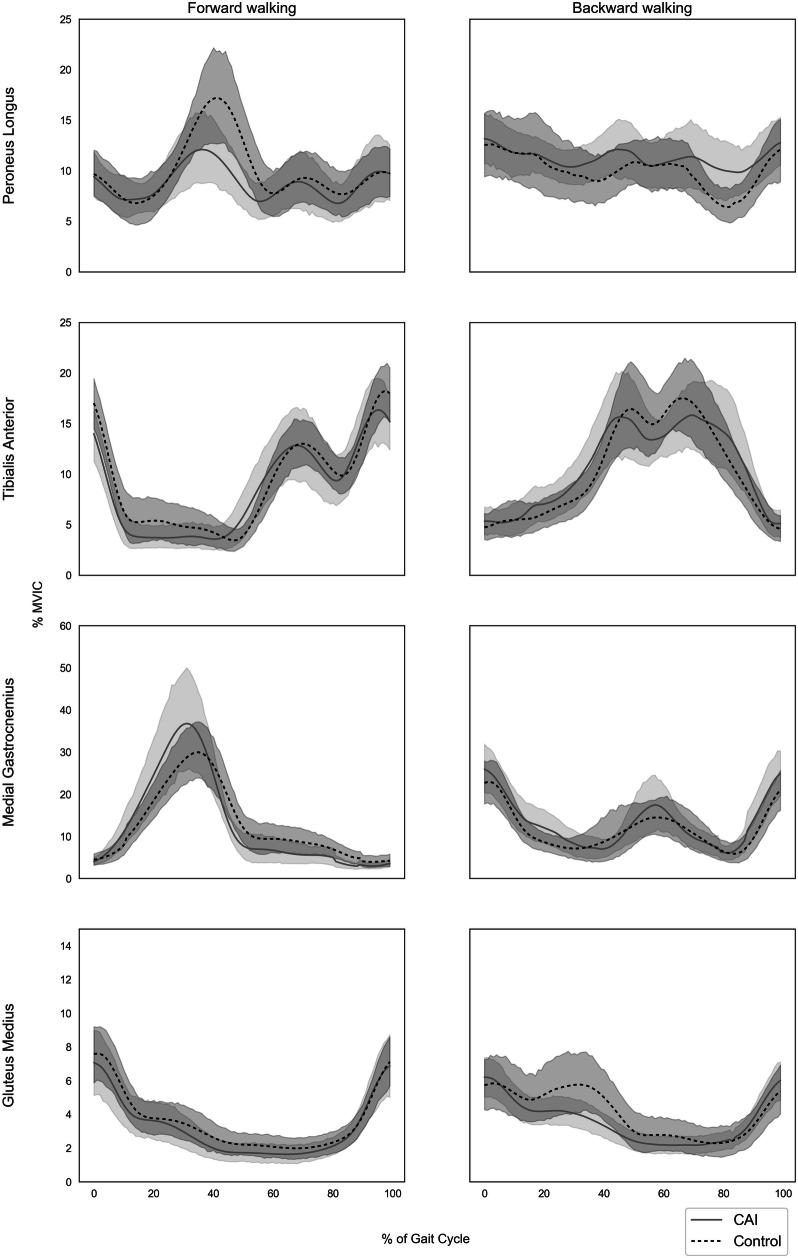
Muscle mean activation as % of maximum voluntary isometric contraction (%MVIC) across the gait cycle. Shaded areas represent 95% confidence intervals

Figure 1 presents the mean EMG activity as %MVIC throughout the gait cycle for each of the tested muscles. As depicted in the figures, overlaps in CIs between the CAI and healthy control groups were consistent throughout the gait cycle in all tested muscles in both walking conditions. Therefore, no significant differences between groups in mean EMG activity can be concluded under BW or FW.

### EMG amplitude variability across the gait cycle

**Fig. 2:**
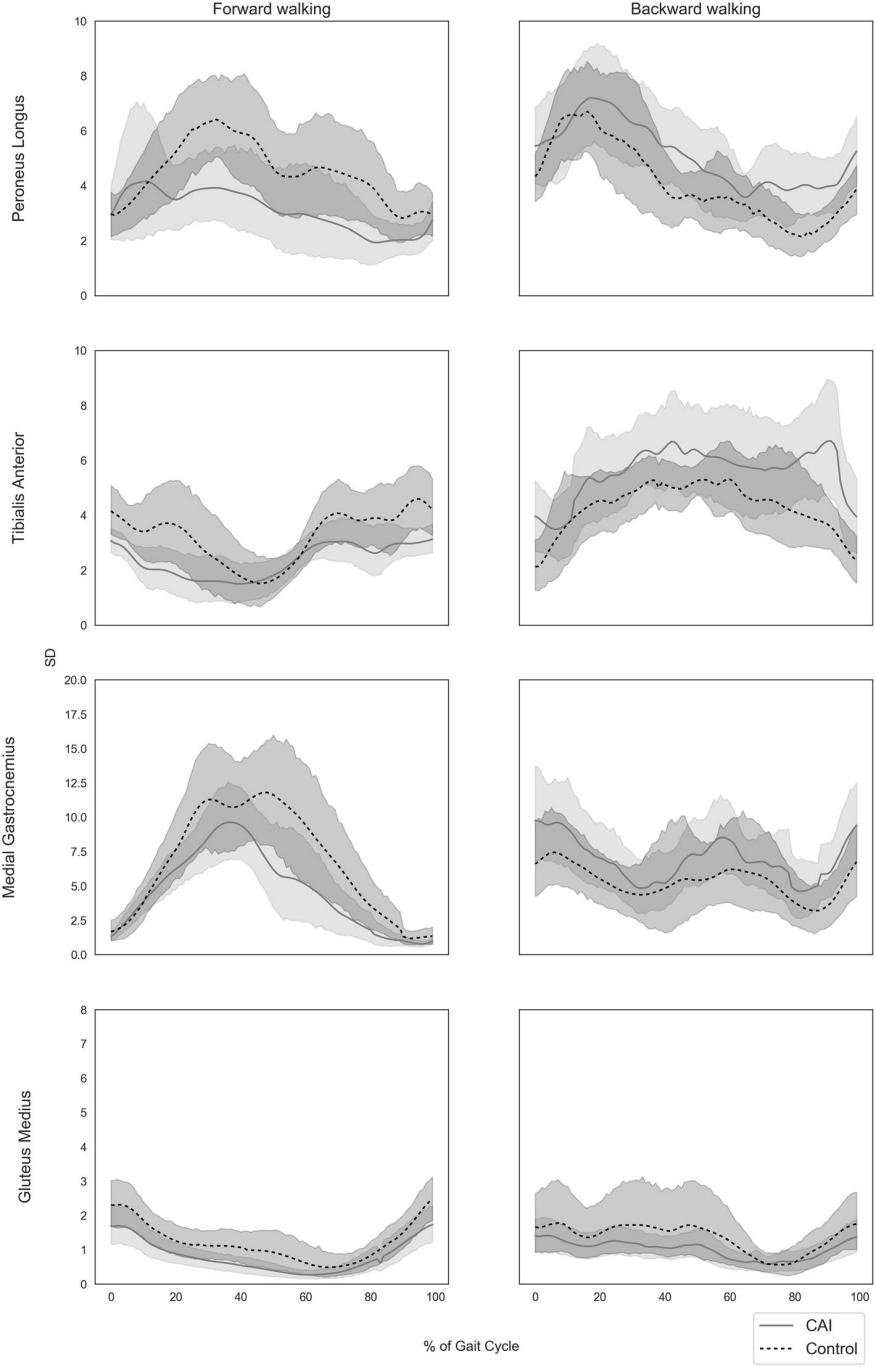
Standard deviations of muscle activity across the gait cycle. Shaded areas represent 95% confidence intervals

Figure 2 shows the mean SD of EMG amplitude throughout the gait cycle for each of the tested muscles. As depicted in the figure, overlaps between the CAI and healthy control groups were consistent throughout the gait cycle in all tested muscles in both walking conditions. Therefore, no significant differences between groups in EMG amplitude variability can be concluded under BW or FW conditions.

### Adaptation to BW

**Fig. 3:**
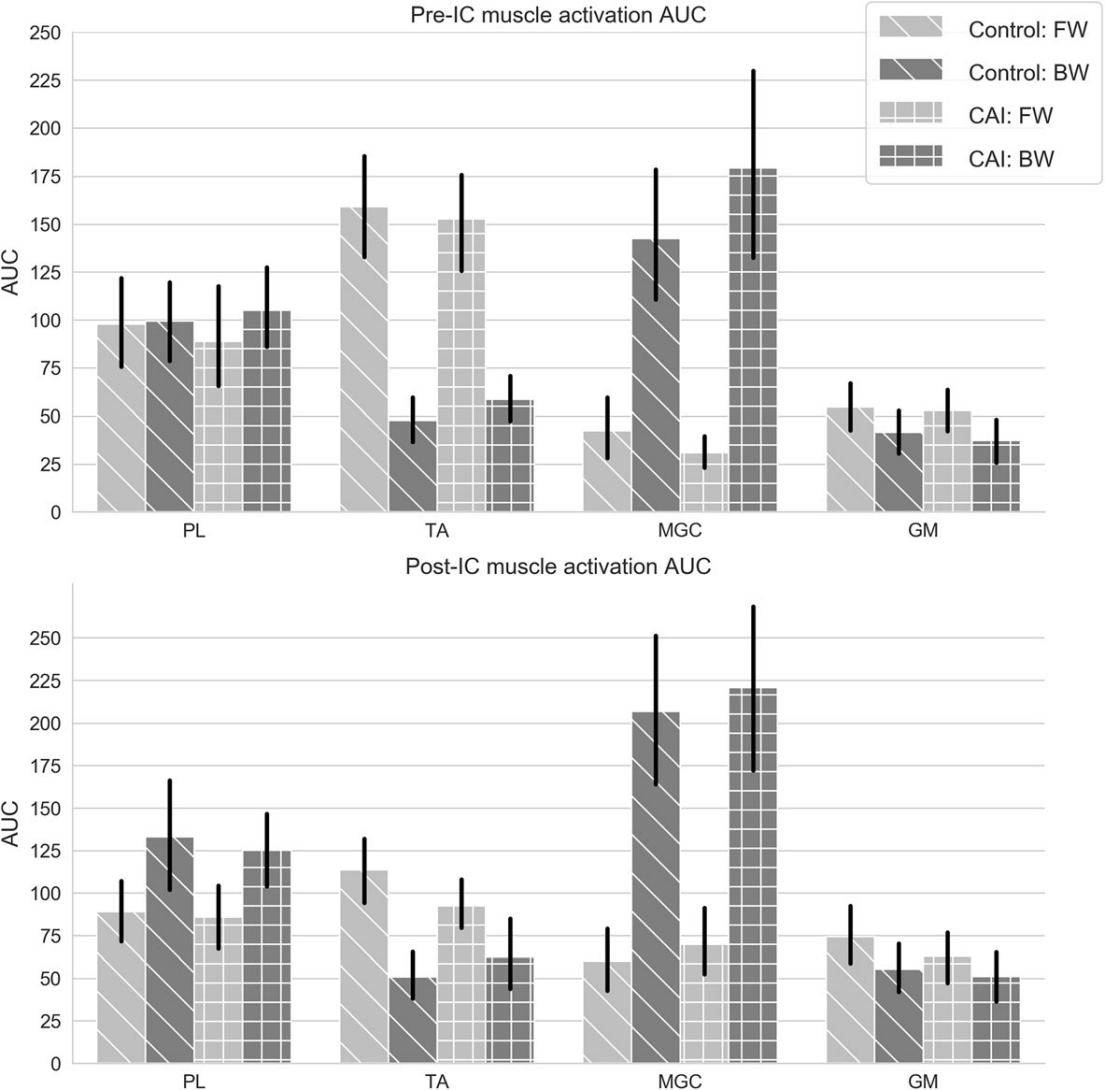
Area under the curve (AUC) for muscle activation as %MVIC at pre-IC and post-IC

Figure 3 shows the pre-IC and post-IC AUC values of all muscles. The results of the linear mixed model analysis for AUC are presented in Table 2. No significant differences between groups were found. Compared to FW, before and after IC under BW condition, decreased tibialis anterior and gluteus medius activation were evident in both groups. In contrast, medial gastrocnemius activation was significantly higher during BW compared to FW in these periods of the gait cycle in both groups. However, no between condition differences were found for peroneus longus activation at pre-IC or at post-IC.

**Table 2:** Results of the linear mixed model analysis for group, condition and interactions. The control group and forward walking are references for group and condition

		Pre-IC		Post-IC	
		Estimate (95% CI)	*p*-value	Estimate (95% CI)	*p*-value
**PL**	Intercept	97.8 (71.0, 124.6)		89.0 (69.5, 108.5)	
	Group	−8.9 (−46.8, 29.0)	0.63	−3.2 (− 30.7, 24.4)	0.82
	Condition	1.6 (−31.0, 34.4)	0.92	23.7 (−6.4, 53.7)	0.12
	Group * Condition	14.9 (−31.3, 61.2)	0.52	15.6 (−26.0, 57.1)	0.45
**TA**	Intercept	159.1 (132.9, 185.3)		113.6 (95.4, 131.8)	
	Group	−6.4 (−43.4, 30.7)	0.73	−20.5 (− 46.3, 5.2)	0.11
	Condition	− 111.3 (− 135.4, −87.2)	< 0.001	− 62.8 (− 84.9, − 40.7)	< 0.001
	Group * Condition	17.2 (− 16.9, 51.3)	0.31	22.4 (−9.0, 53.8)	0.16
**MGC**	Intercept	42.3 (28.8, 55.8)		60.2 (40.2, 80.2)	
	Group	−11.4 (−30.4, 7.7)	0.23	9.9 (−18.4, 38.2)	0.48
	Condition	100.1 (63.7, 136.6)	< 0.001	146.7 (105.8, 187.5)	< 0.001
	Group * Condition	36.2 (−16.2, 88.6)	0.17	4.3 (−53.5, 62.0)	0.88
**GM**	Intercept	54.6 (42.0, 67.2)		74.5 (56.9, 92.0)	
	Group	−1.9 (− 19.8, 16.0)	0.83	−11.6 (−36.4, 13.1)	0.35
	Condition	−13.0 (− 22.9, − 3.2	0.01	−19.0 (− 33.6, −4.5)	0.01
	Group * Condition	−2.3 (− 16.2, 11.6)	0.73	7.3 (− 13.2, 27.9)	0.47

* *CAI* chronic ankle instability, *IC* initial contact, *PL* peroneus longus, *TA* tibialis anterior, *MGC* medial gastrocnemius, *GM* gluteus medius

**Fig. 4:**
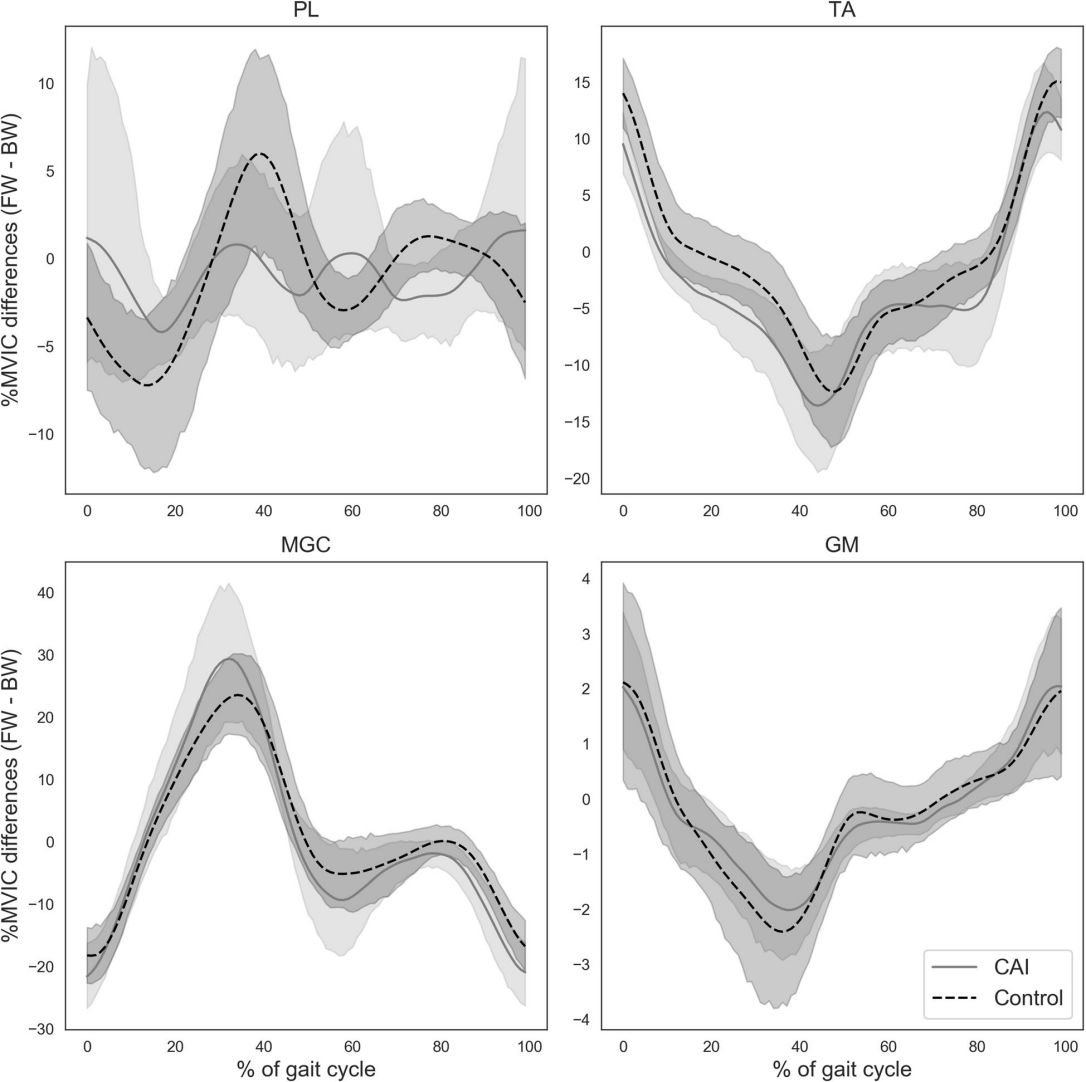
Differences in muscle mean activation as % of maximum voluntary isometric contraction (%MVIC) between forward and backward walking, across the gait cycle. Shaded areas represent 95% confidence intervals

The differences in EMG amplitudes between FW and BW are presented in Fig. 4. As depicted in the figure, overlaps between the CAI and healthy control groups were consistent throughout the gait cycle in all tested muscles.

## Discussion

The purpose of this study was to determine whether leg muscle EMG amplitude and activation variability during BW differs between groups with and without CAI. Major differences were found between BW and FW. However, in contrast to our hypothesis, no between group differences were evident. This may indicate that participants with CAI and healthy controls use similar motor adaptations to BW.

To the best of our knowledge, no previous study has fully assessed EMG patterns during BW in people with CAI. Therefore, comparison is only possible with earlier reports that evaluated EMG during FW in this population. In a study that assessed EMG amplitude of the peroneus longus, tibialis anterior, lateral gastrocnemius, rectus femoris, biceps femoris, and gluteus medius muscles, no differences were noted between CAI and healthy groups at either 100 msec before or 200 msec after IC, during shod, treadmill walking at fixed 4.8 km/h speed. In this study, the area under the RMS curve was normalized to quiet standing [12]. Similarly, a recently published study used equivalent methods to analyze EMG amplitudes of peroneus longus, tibialis anterior, medial gastrocnemius, and gluteus medius muscles during shod, treadmill FW at self-selected or 120% of self-selected speed. The authors reported no group differences between people with CAI and individuals who had an ankle sprain but learned to cope and returned to pre-injury levels of function [34]. Likewise, several studies that evaluated EMG amplitude of leg muscles during dynamic tasks such as jump landings also reported no differences between participants with CAI and matched healthy controls [35–37]. For example, a recently published study by Simpson and colleagues [37] recorded muscle activity from the peroneus longus and peroneus brevis, tibialis anterior, and medial gastrocnemius, during pre- to post unexpected and expected single leg drop-landings. No differences in average EMG amplitude were found between individuals with CAI and healthy controls.

Consisted with the reports that evaluated EMG amplitude and found no differences between subjects with and without CAI, some evidence suggests no difference in muscle activation variability between these populations. Kautzky et al. [14] studied the variability in muscle function during FW using several methods as mentioned above. There were no significant differences between CAI and healthy controls groups in the variability of activation of the peroneus longus, tibialis anterior, lateral gastrocnemius, rectus femoris, biceps femoris, and gluteus medius across the stride cycle. Therefore, the present findings and the above-mentioned studies may provide evidence that similar motor control strategies are used by individuals with and without CAI.

Yet, conflicting results were reported in other studies. Louwerens et al. [13] evaluated CAI and healthy subjects during shod, treadmill walking at self-selected speed and at 50% of self-selected speed. EMG linear envelope transformed signals were normalized to the to the highest recorded peak during the gait cycle. Subjects with CAI displayed significant increase in tibialis anterior amplitude during the stance phase under both walking conditions. Delahunt et al. [9] observed increased area under the RMS curve of peroneus longus activity 40-80 msec after IC during barefoot, treadmill FW at 4 km/h speed in participants with CAI, with normal activity of this muscle pre-IC. Koldenhoven et al. [11] reported that during shod, treadmill FW at 4.8 km/h, participants with CAI had decreased tibialis anterior activity and significantly increased peroneus longus, medial gastrocnemius, and gluteus medius activity pre-IC, with no difference post-IC. In another study by this group, decreased stride-to-stride variability in both the tibialis anterior and peroneus longus activation was identified post-IC in individuals with CAI [15].

Due to the large discrepancies between studies, it seems that no sound conclusion can be drawn regarding leg muscle EMG amplitude and activation variability during gait and dynamic tasks among individuals with CAI. However, several explanations are proposed for the inconsistencies observed between studies. Some discrepancies might be due to the heterogeneity of the CAI population. The updated paradigm of CAI suggests that there is a list of impairments that people with CAI as a group, are likely to demonstrate; however, each individual may present certain clinical and performance outcomes that are affected by personal and environmental factors [38]. Specifically, all the CAI participants in the current study met established standards for the diagnosis [5]. However, according to the Ankle Instability Instrument, only 3 of 16 participants reported that they felt unstable while walking on a flat surface. Previous studies have shown that only very complex walking situations, such as walking with a cognitive dual task, may differentiate individuals with CAI from controls [39, 40]. Indeed, comparing some previous studies [9, 11, 15] with the present study, during self-selected FW there were no differences between CAI and matched controls. This may indicate that the individuals with CAI that were investigated, no functional impairments while performing a simple ambulation task. These findings are consisted with previous data showing that walking under self-selected speed may not challenge the sensorimotor control of subjects with CAI [39]. Thus, it is possible that while BW on a treadmill required some level of adaptation from both groups, it was not challenging enough to discriminate their gait performance. Assessments with more challenging tasks, such as BW with dual task and BW at fast speeds may be more appropriate for testing gait impairments in individuals with CAI. As previously suggested [38], it is recommended to identify patient-specific complaints and impairments to guide the development of assessments and interventions in CAI.

Another aspect that could explain the variation in findings between studies is related to data collection. The reference for EMG normalization differs significantly between studies; thus, it is difficult to conclude whether the differences were due to the group or to the normalization methods. Furthermore, while some studies evaluated discrete EMG signals [9, 12], others have analyzed continuous data from the entire gait cycle [34]. In addition, systematic reviews indicated significant differences in muscle activity during barefoot and shod walking and running [41, 42]. In the present study, we evaluated both continuous and discrete (Pre- and Post- IC) EMG data, which were collected while participants walked barefoot at a self-selected speed; thus, the results should be interpreted with caution when compared to other studies.

Although BW did not distinguish between groups, it influenced muscle activation in both groups compared to FW. This finding is agreement with previous studies that reported changes in lower limb muscle activation patterns during BW, as compared to FW [18, 20, 23, 27]. Consistent with previous research, the gastrocnemius muscle was mostly active during early stance in BW gait, whereas the tibialis anterior was active during early stance in FW [27]. The greater activation of the gluteus medius muscle observed in the present study during BW may be related to an effort to provide more control during pre- and post- IC. It was shown that during BW, as compared to FW, the pelvis is less stable vertically [43]. This decrease in pelvic stability may be the cause of the increased activity of the gluteus medius muscle.

Interestingly, a systematic review of studies that assessed the effects of CAI on muscle activity during FW concluded that while some studies observed significant differences between the CAI and the control groups, and others did not, it seems that increased activity of the peroneus longus pre-IC is often found in participants with CAI [44]. However, the present study did not find differences in peroneus longus activation either between groups or between walking conditions. Therefore, based on these results, it is unlikely that BW can be used as specific training to improve control over the peroneus longus in CAI. Yet, current evidence suggests that BW uses additional elements, presumably supraspinal, in addition to a common FW spinal drive [17, 18]. The significant alterations in muscle activity during BW in both groups, may support this notion. Thus, it may be used as a training technique in order to stress the sensorimotor system. There are currently no evidence-based recommendations for CAI gait retraining. Therefore, future research should assess the effect of BW training on individuals with CAI.

There are large discrepancies in EMG parameters between studies that assessed muscle activity during gait in the CAI population. Based on previous research, in the present investigation we examined both EMG amplitude and AUC, which are two of the most commonly used outcomes [44]. Furthermore, the time and duration of activation were not analyzed directly. Yet, the overlaps between groups in the amplitude of the tested muscles throughout the gait cycle may suggest that the time and duration of muscle activity do not differ between groups. Finally, to date, only two studies reported variability of muscle function during walking in individuals with CAI [14, 15]. The variability of EMG amplitude that was also analyzed in the current study may contribute to the understanding of the consistency of motor pattern in CAI population. It is recommended that the methods applied in this study should be used in future investigations that evaluate gait in this population. Such studies should confirm the present results with more participants, including subgroups of people with CAI who have varied levels of impairment.

## Conclusions

Backward gait training is becoming a popular treatment method for people with musculoskeletal disorders. Therefore, it is important to understand how the application of BW affects motor control among individuals with CAI. This study indicates that, compared to FW, participants with CAI and healthy controls demonstrate significant changes in mean EMG activation and activation variability in leg muscles during BW. However, there are no differences between groups. Due to the heterogeneity of the CAI population, the results should be interpreted with caution. Future investigations that evaluate motor control during BW with larger and varied cohorts of people with CAI are warranted.

## Data Availability

The datasets used and analyzed during the current study are available from the corresponding author on reasonable request.

## Abbreviations

AUC: Area under the curve
BW: Backward walking
CAI: Chronic ankle instability
CI: Confidence interval
COV: Coefficient of variation.
EMG: Electromyography
FW: Forward walking
GM: Gluteus medius
IC: Initial contact
LT: Left
M: Mean
MGC: Medial gastrocnemius
MVIC: Maximum voluntary isometric contraction
PL: Peroneus longus
RMS: Root mean square
RT: Right
SD: Standard deviation
TA: Tibialis anterior
